# Weight-adjusted waist index and all-cause and cardiovascular mortality in chronic kidney disease: A cohort study with mediation analysis of systemic inflammation

**DOI:** 10.1097/MD.0000000000050613

**Published:** 2026-09-04

**Authors:** Hao Zhou, Xue Zhang, Wanjun Zhang, Guanping Liu, Hairong Wang

**Affiliations:** a Department of Nephrology, Guanghan Hospital of Traditional Chinese Medicine, Deyang, Sichuan, China; b Department of Emergency, Deyang People’s Hospital, Deyang, Sichuan, China; c Xi’an Jiaotong University, School of Public Policy and Administration, Xi’an, Shaanxi, China.

**Keywords:** adjusted waist index, CKD, mortality, neutrophil-to-lymphocyte ratio, weight

## Abstract

The weight-adjusted waist index (WWI), a recently proposed marker reflecting central fat distribution independent of body weight, has emerged as a potential indicator of metabolic and cardiovascular risk. Considering the high burden of inflammation and metabolic disturbance in chronic kidney disease (CKD), we examined whether elevated WWI is associated with all-cause and cardiovascular disease (CVD) mortality among CKD patients and whether systemic inflammation, assessed by the neutrophil-to-lymphocyte ratio (NLR), mediates this relationship. Data were obtained from the National Health and Nutrition Examination Survey 1999 to 2018 cycles. WWI was calculated based on anthropometric measurements. Weighted multivariate Cox proportional hazards models were applied to examine the associations of WWI with mortality. Robustness was verified through WWI quartile-based survival curves and subgroup analyses. Mediation analysis was used to evaluate the indirect effect of NLR on the WWI–mortality relationship. A total of 5582 CKD participants were included. WWI levels were significantly and independently associated with increased risks of all-cause mortality (adjusted hazard ratio = 1.17, 95% confidence interval (CI): 1.09–1.26, *P* < .001) and CVD mortality (adjusted hazard ratio = 1.24, 95% CI: 1.09–1.41, *P* = .001). WWI showed a linear positive relationship with both outcomes (nonlinearity *P* > .05). Mediation analysis indicated that NLR exerted a modest but significant indirect effect on all-cause mortality (*β* = −9.94, 95% CI:−20.90–−2.85, *P* < .001), explaining 6.66% of the total effect. Elevated WWI was independently associated with higher risks of all-cause and CVD mortality in CKD. NLR partially mediated these associations. Therefore, assessing both central adiposity and inflammatory burden may aid in identifying high-risk CKD patients and guide clinical strategies for improving long-term outcomes.

## 1. Introduction

Chronic kidney disease (CKD) is a common condition contributing to reduced health-related quality of life and increased mortality. It is characterized by decreased glomerular filtration rate and/or increased urinary albumin excretion, and constitutes a substantial global public health burden.^[1–3]^ In the United States (U.S.), approximately 14% of adults are affected by CKD.^[4]^ With the rising prevalence of obesity and diabetes, the prevalence of CKD is projected to reach 29 to 68% by 2030.^[5]^

Obesity is characterized by excessive accumulation of adipose tissue. According to World Health Organization, obesity is defined as a body mass index (BMI) ≥ 30 kg/m^2^, and overweight as a BMI between 25 and 30 kg/m^2^.^[6]^ However, BMI has limitations in accurately assessing obesity, as individuals with similar BMI values may differ significantly in body composition and fat distribution.^[7]^ Moreover, BMI fails to capture abdominal adiposity, whereas waist circumference (WC) better reflects it yet still lacks discrimination between subcutaneous and visceral fat.^[8,9]^ The weight-adjusted waist index (WWI) integrates the advantages of WC while overcoming the limitations of BMI, offering a more accurate assessment of fat and muscle mass. Independent of body weight, WWI reliably reflects abdominal obesity and better measures fat and muscle levels than BMI, WC, or the visceral adiposity index.^[10–13]^ In American adults, WWI was independently associated with cardiovascular disease (CVD).^[14]^ Similarly, among individuals with hyperlipidemia, WWI showed a positive linear association with mortality.^[15]^ These findings indicate that WWI may serve as a reliable indicator of obesity-related risk and metabolic health.^[16]^

However, the relationship between WWI and mortality in CKD remains uncertain, including whether it is mediated by the neutrophil-to-lymphocyte ratio (NLR). Accordingly, we assessed the association of WWI with all-cause and cardiovascular mortality in CKD and examined NLR as a potential mediator.

## 2. Methods

### 2.1. Study population

Data were drawn from National Health and Nutrition Examination Survey (NHANES) 1999 to 2018, a nationally representative survey of the U.S. population. The primary analysis was restricted to the NHANES 1999 to 2016 cycles because linked mortality data were available only through December 31, 2019, and inclusion of the 2017 to 2018 cycles would have resulted in substantially shorter follow-up for mortality assessment. Adults (≥ 20 years) meeting the Kidney Disease: Improving Global Outcomes criteria for CKD were included; those lacking anthropometric, mortality, or medical data were excluded. A sensitivity analysis including the NHANES 1999 to 2018 cycles was additionally performed to assess the robustness of the findings. However, NHANES does not provide reliable information to identify dialysis status; therefore, patients with CKD stage 5 requiring dialysis could not be separately classified in this study.

### 2.2. Outcomes and definitions

Primary outcomes included all-cause and CVD mortality. Mortality data were linked to the National Death Index through probabilistic matching. CVD mortality was identified using International Classification of Diseases, 10th Revision codes I00 to I09, I11, I13, I20 to I51, and I60 to I69. Follow-up continued until death or December 31, 2019. CKD was defined per Kidney Disease: Improving Global Outcomes guidelines as estimated glomerular filtration rate (eGFR) < 60 mL/minute/1.73 m^2^ or urinary albumin-to‑creatinine ratio ≥ 30 mg/g,^[17]^ with eGFR estimated using the Chronic Kidney Disease Epidemiology Collaboration equation.^[18]^

Measurement of WWI and NLR: WWI (cm/√kg) = WC (cm)/√body weight (kg). NLR was calculated by dividing absolute neutrophil count by absolute lymphocyte count from automated hematology tests (Beckman Coulter method).

Covariates included: demographics: age, sex, race, marital status, education, poverty-to-income ratio, smoking and drinking status; physical measures: BMI; comorbidities: hypertension, diabetes, dyslipidemia, and CVD. Definitions followed NHANES standards: BMI categories: underweight < 18.5, normal 18.5 to 24.9, overweight 25 to 29.9, obese ≥ 30 kg/m^2^; diabetes: history or medication use, hemoglobin A1c ≥ 6.5%, fasting plasma glucose ≥ 126 mg/dL, or 2-hour plasma glucose ≥ 200 mg/dL; hypertension: history or 2-hour plasma glucose ≥ 140 mm Hg or diastolic blood pressure ≥ 90 mm Hg; dyslipidemia: total cholesterol ≥ 200 mg/dL, triglyceride ≥ 150 mg/dL, low‑density lipoprotein cholesterol ≥ 130 mg/dL, or high‑density lipoprotein cholesterol ≤ 50 mg/dL in women (≤ 40 mg/dL in men)^[19]^ or lipid-lowering therapy; CVD: coronary heart disease, heart failure, myocardial infarction, stroke, or angina.

### 2.3. Statistical analysis

Statistical analyses were performed using R (version 4.5.1; R Foundation for Statistical Computing, Vienna, Austria). In accordance with the NHANES analytic guidelines on the use of survey weights, all analyses incorporated appropriate sampling weights, clustering, and stratification to ensure accurate variance estimation and nationally representative estimates for the U.S. noninstitutionalized civilian population, particularly individuals with CKD. For the primary analysis, participants from the 1999 to 2016 cycles were followed until death or December 31, 2019. Weighted Cox regression estimated hazard ratios (95% confidence intervals [CIs]) for WWI with all-cause and CVD mortality. Linearity was assessed using restricted cubic splines. Subgroup analyses stratified by age, sex, BMI, comorbidities, and race verified robustness. The full NHANES 1999 to 2018 period was examined as a sensitivity analysis, and the results were consistent with the primary analysis. Additional sensitivity analyses were performed by excluding participants with eGFR < 15 mL/minute/1.73 m^2^, and subgroup analyses were further conducted according to baseline renal function (eGFR < 45, 45–60, and ≥ 60 mL/minute/1.73 m^2^) to evaluate the robustness of the findings. Bayesian mediation models assessed indirect effects of NLR on the WWI–mortality association. *P* < .05 was considered statistically significant.

### 2.4. Ethical approval

This study was based on publicly available, de-identified data from the NHANES. The NHANES protocol was approved by the National Center for Health Statistics Ethics Review Board, and all participants provided written informed consent at the time of data collection. Therefore, no additional ethical approval was required for this secondary analysis.

## 3. Results

### 3.1. Characteristics of included patients

Among 5582 participants (median follow-up 8.5 years) (Fig. S1, Supplemental Digital Content 1), 3347 (59.96%) survived and 2235 (40.04%) died (Table 1). Compared with survivors, non-survivors were significantly older and had higher levels of urinary albumin-to-creatinine ratio and NLR, but lower serum albumin, hemoglobin, and eGFR. They were more likely to be male, hypertensive, diabetic, dyslipidemic, and to have preexisting CVD. In addition, a more advanced distribution of CKD stages was observed in the non-survivor group, with a higher proportion of patients in CKD stages G3b–G5. Socioeconomic status was also poorer among non-survivors, with lower education, income, and higher rates of being single or previously married.

**Table 1:** Baseline characteristics of the study population.

Variable	Total (n = 5582)	Survivors (n = 3347)	Non-Survivors (n = 2235)	*P*
Age, years, Mean (SE)	59.45 (0.36)	53.34 (0.43)	71.28 (0.37)	**< .001**
Sex, n (%)				**< .001**
Male	2669 (42.96)	1470 (40.86)	1199 (47.01)	
Female	2913 (57.04)	1877 (59.14)	1036 (52.99)	
Race, n (%)				**< .001**
Mexican American	860 (6.75)	610 (8.42)	250 (3.51)	
Non-Hispanic White	2720 (69.72)	1335 (64.97)	1385 (78.90)	
Non-Hispanic Black	1345 (13.54)	888 (14.64)	457 (11.41)	
Other	657 (10.00)	514 (11.97)	143 (6.17)	
Education, n (%)				**< .001**
< High School	1927 (24.87)	1003 (20.41)	924 (33.51)	
High School or some College	1348 (25.75)	808 (25.18)	540 (26.85)	
College or Above	2307 (49.38)	1536 (54.41)	771 (39.64)	
Marital status, n (%)				**< .001**
Married or living with a partner	3089 (59.19)	1967 (62.56)	1122 (52.66)	
Single (widowed/divorced/separated)	1958 (30.75)	960 (24.84)	998 (42.21)	
Never married	535 (10.06)	420 (12.60)	115 (5.13)	
PIR, n (%)				**< .001**
< 1.3	1874 (25.66)	1112 (24.75)	762 (27.42)	
1.3–3.5	2349 (41.31)	1337 (38.39)	1012 (46.97)	
≥ 3.5	1359 (33.03)	898 (36.87)	461 (25.61)	
Drinking, n (%)				**< .001**
No	1991 (32.64)	1131 (29.29)	860 (39.14)	
Yes	3591 (67.36)	2216 (70.71)	1375 (60.86)	
Smoking, n (%)				**< .001**
Never	2727 (48.97)	1775 (52.24)	952 (42.64)	
Former	1873 (32.45)	939 (28.01)	934 (41.05)	
Now	982 (18.58)	633 (19.75)	349 (16.31)	
BMI group, (kg/m^2^) n (%)				**.006**
Underweight (< 18.5)	96 (2.11)	53 (2.14)	43 (2.05)	
Normal (18.5–25)	1319 (25.09)	707 (23.84)	612 (27.53)	
Overweight (25–30)	1812 (30.32)	1037 (29.56)	775 (31.77)	
Obese (≥ 30)	2355 (42.48)	1550 (44.46)	805 (38.65)	
HTN, n (%)				**< .001**
No	1591 (34.21)	1219 (42.92)	372 (17.34)	
Yes	3991 (65.79)	2128 (57.08)	1863 (82.66)	
DM, n (%)				**< .001**
No	3470 (67.64)	2194 (72.40)	1276 (58.43)	
Yes	2112 (32.36)	1153 (27.60)	959 (41.57)	
Hyperlipidemia, n (%)				**< .001**
No	974 (17.95)	611 (19.61)	363 (14.75)	
Yes	4608 (82.05)	2736 (80.39)	1872 (85.25)	
CVD, n (%)				**< .001**
No	4109 (76.47)	2780 (85.07)	1329 (59.82)	
Yes	1473 (23.53)	567 (14.93)	906 (40.18)	
CKD stage, n (%)				**< .001**
G1-G2 (eGFR ≥ 60mL/min/1.73m^2^ + UACR ≥ 30mg/g	3077 (58.81)	2185 (67.49)	892 (42.18)	
G3a (45–59mL/min/1.73m^2^)	1662 (28.69)	836 (25.00)	826 (35.73)	
G3b (30–44mL/min/1.73m^2^)	606 (9.24)	251 (5.92)	355 (15.59)	
G4 (15–29mL/min/1.73m^2^)	172 (2.39)	52 (1.13)	120 (4.78)	
G5 (< 15mL/min/1.73m^2^)	65 (0.88)	23 (0.45)	42 (1.72)	
Serum albumin (g/L) , Mean (SE)	41.98 (0.07)	42.38 (0.09)	41.21 (0.09)	**< .001**
Hb (g/dL) , Mean (SE)	13.98 (0.04)	14.06 (0.04)	13.81 (0.05)	**< .001**
Daily energy intake,2-day average (kcal/day) , Mean (SE)	1888.89 (14.59)	1972.23 (18.08)	1727.60 (22.75)	**< .001**
UACR, (mg/g), Mean (SE)	184.02 (10.08)	144.56 (9.42)	260.39 (21.85)	**< .001**
eGFR, (mL/min/1.73 m^2^), Mean (SE)	76.04 (0.51)	82.97 (0.64)	62.64 (0.70)	**< .001**
NLR, Mean (SE)	2.54 (0.03)	2.35 (0.03)	2.91 (0.06)	**< .001**

BMI = body mass index, CKD = chronic kidney disease, CVD = cardiovascular disease, DM = diabetes mellitus, eGFR = estimated glomerular filtration rate, Hb = hemoglobin, HTN = hypertension, n = number of patients, NLR = neutrophil-to-lymphocyte ratio, PIR = poverty-to-income ratio, SE = standard error, UACR = urine albumin-to-creatinine ratio.

### 3.2. WWI and risk of all-cause and CVD mortality

**Figure 1.**
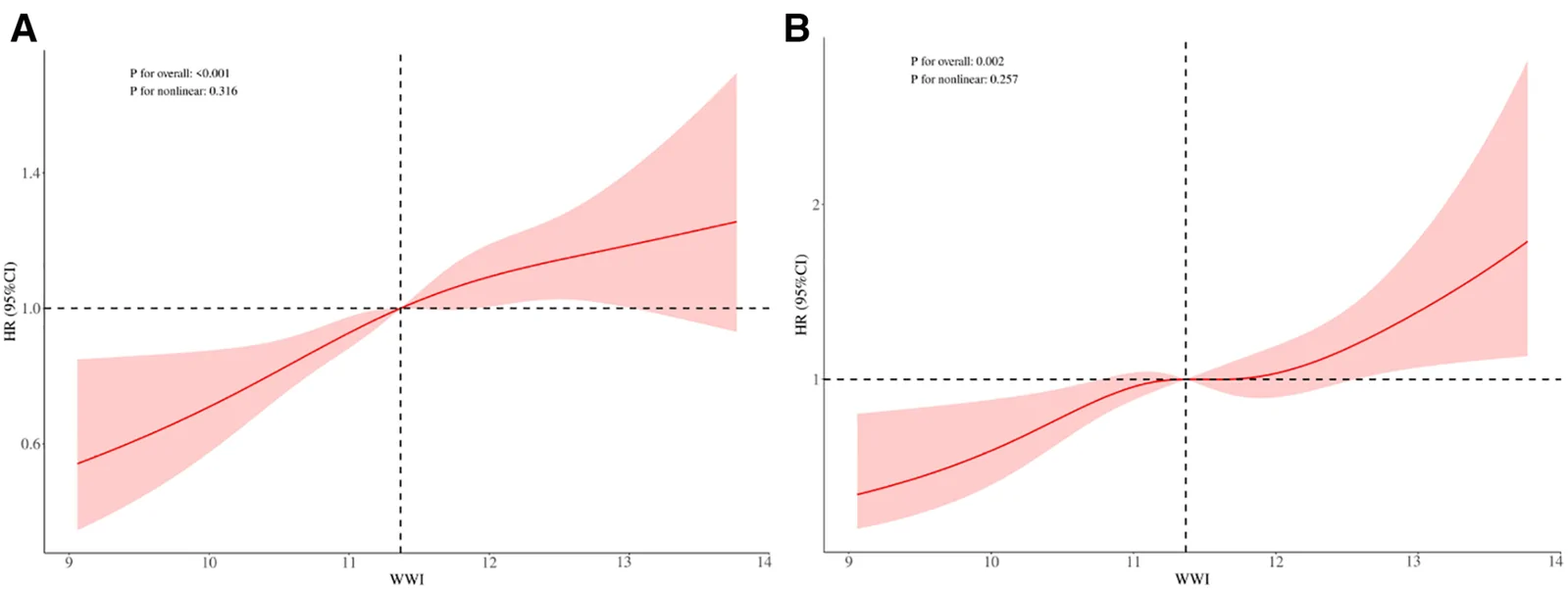
Association between WWI and (A) all-cause and (B) CVD mortality in patients with CKD, based on the fully adjusted Cox proportional hazards model. CI = confidence interval, CKD = chronic kidney disease, CVD = cardiovascular disease, HR = hazard ratio, WWI = weight-adjusted waist index.

**Figure 2.**
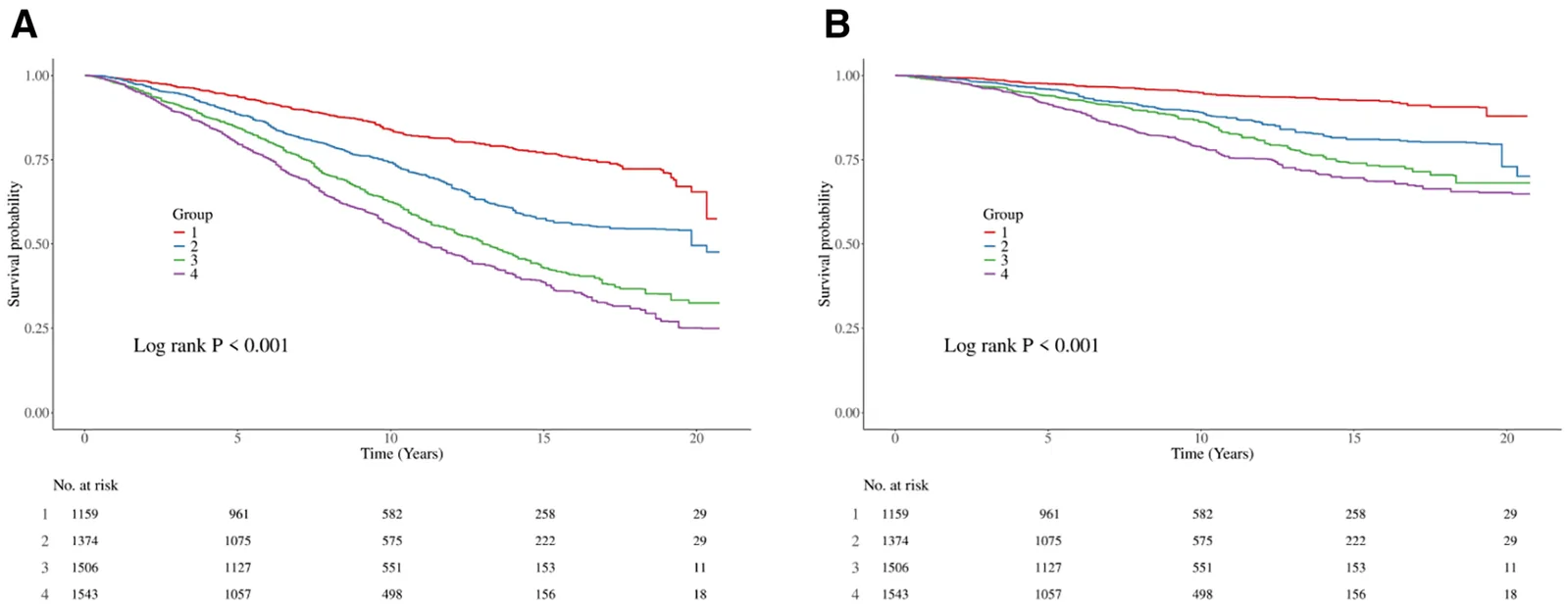
KM survival curves of (A) all-cause and (B) CVD mortality according to WWI quartiles among patients with CKD. CKD = chronic kidney disease, CVD = cardiovascular disease, KM = Kaplan–Meier, WWI = weight-adjusted waist index.

**Figure 3.**
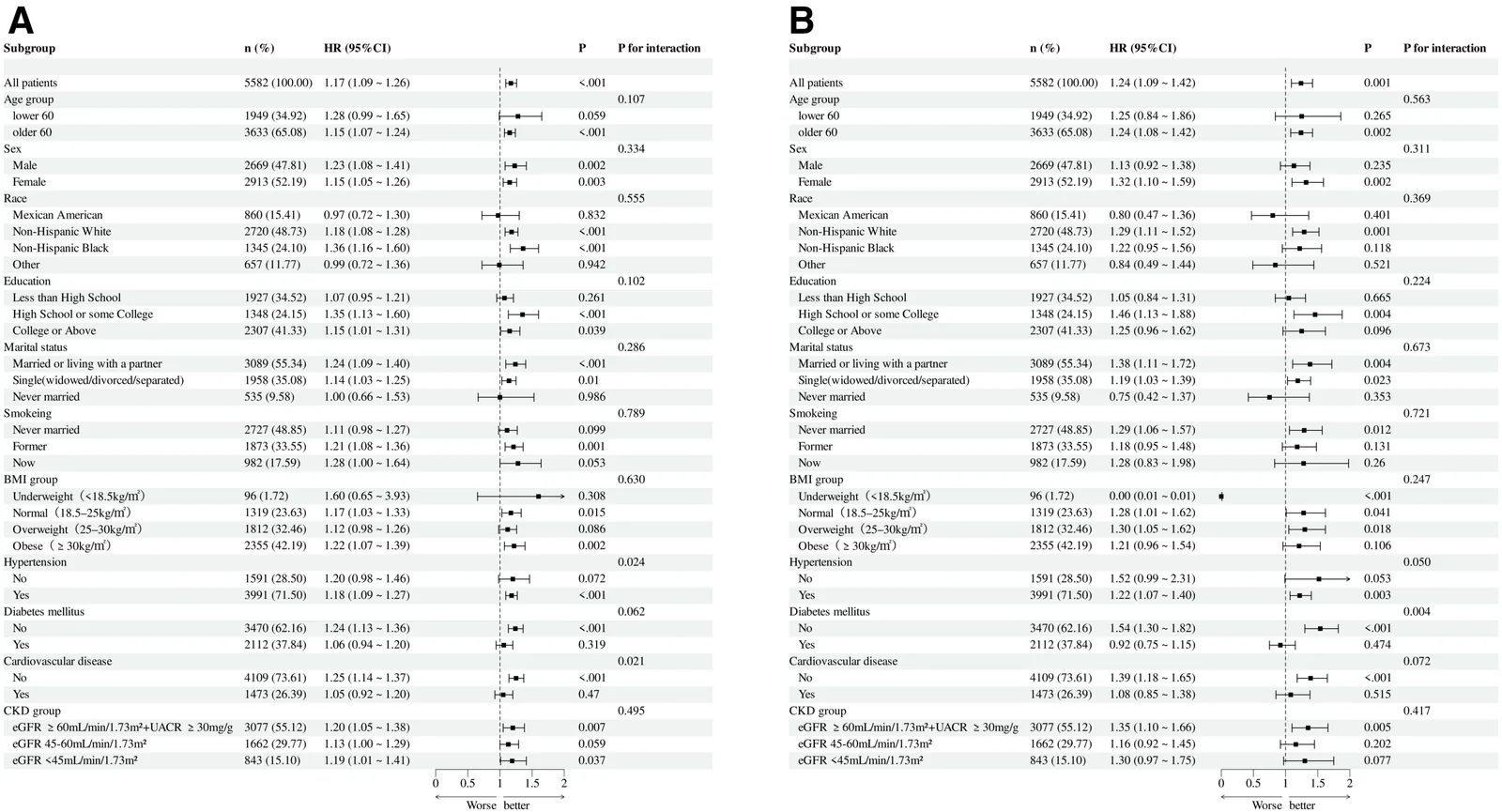
Subgroup analyses of WWI on (A) all-cause and (B) CVD mortality in patients with CKD, based on the fully adjusted Cox proportional hazards model. BMI = body mass index, CI = confidence interval, CKD = chronic kidney disease, CVD = cardiovascular disease, eGFR = estimated glomerular filtration rate, HR = hazard ratio, n = number of patients, WWI = weight-adjusted waist index.

Multivariable Cox proportional hazards indicated that each 1-unit increase in WWI was associated with 17% higher all-cause mortality risk adjusted hazard ratio (aHR) = 1.17, 95% CI: 1.09–1.26, *P* < .001) and 24% higher CVD mortality risk (aHR = 1.24, 95% CI: 1.09–1.41, *P* = .001). Compared with the lowest quartile (Q1), the highest quartile (Q4) had significantly elevated risks for both all-cause (aHR = 1.28, 95% CI: 1.08–1.52, *P* = .005) and CVD mortality (aHR = 1.39, 95% CI: 1.03–1.89, *P* = .033) (Table 2 and 3). Nonlinear tests were nonsignificant (*P* > .05), indicating a linear trend (Figs. 1A and B). Kaplan–Meier curves stratified by WWI quartiles demonstrated progressively lower survival rates with increasing WWI (log-rank *P* < .001) (Figs. 2A and 2B). Subgroup analyses revealed significant effect modification(Figs. 3A and 3B). The WWI-associated mortality risk was higher in men (aHR = 1.23, 95% CI: 1.08–1.41 for all-cause; aHR = 1.33, 95% CI: 1.11–1.59 for CVD) and more pronounced among younger patients. In “low-risk” subgroups without hypertension, diabetes, or CVD, WWI remained a strong predictor of mortality. The associations were consistent across BMI categories and racial groups, indicating the generalizability of WWI-related risks across diverse clinical and demographic profiles. These results highlight the heterogeneity of central obesity-related mortality risk in CKD. Additional sensitivity analyses excluding participants with eGFR < 15 mL/minute/1.73 m^2^ and analyses based on the full NHANES 1999 to 2018 cohort yielded consistent results (Table S1, Supplemental Digital Content 2). Furthermore, subgroup analyses stratified by baseline renal function (eGFR < 45, 45–60, and ≥ 60 mL/minute/1.73 m^2^) showed no significant interaction (all *P* for interaction > .05), indicating that the association between WWI and mortality remained consistent across different levels of kidney function (Figs. 3A and 3B).

**Table 2:** Association between WWI and all-cause mortality in CKD.

Variables	Model 1		Model 2		Model 3	
	HR (95% CI)	*P*	HR (95% CI)	*P*	HR (95% CI)	*P*
WWI (continuous)	1.79 (1.67–1.91)	**< .001**	1.28 (1.19–1.38)	**< .001**	1.17 (1.09–1.26)	**< .001**
WWI quartiles						
Q1	1.00 (Reference)		1.00 (Reference)		1.00 (Reference)	
Q2	2.33 (1.73–3.15)	**< .001**	1.17 (0.97–1.40)	.102	1.11 (0.92–1.33)	.269
Q3	3.21 (2.44–4.21)	**< .001**	1.43 (1.20–1.71)	**< .001**	1.33 (1.13–1.58)	**< .001**
Q4	4.34 (3.27–5.76)	**< .001**	1.50 (1.26–1.78)	**< .001**	1.28 (1.08–1.52)	**.005**

Model 1: Crude.

Model 2: adjust: sex, age, race, education, marital status, PIR, drinking, smoking, BMI, daily energy intake (2-day average).

Model 3: adjust: sex, age, race, education, marital status, PIR, drinking, smoking, BMI, daily energy intake (2-day average), HTN, DM, hyperlipidemia, CVD, serum albumin, Hb, UACR, eGFR.

BMI = body mass index, CI = confidence interval, CKD = chronic kidney disease, CVD = cardiovascular disease, DM = diabetes mellitus, eGFR = estimated glomerular filtration rate, HR = hazard ratio, HTN = hypertension, PIR = poverty-to-income ratio, UACR = urine albumin-to-creatinine ratio, WWI = weight-adjusted waist index.

**Table 3:** Association between WWI and CVD mortality in CKD.

Variables	Model 1		Model 2		Model 3	
	HR (95% CI)	*P*	HR (95% CI)	*P*	HR (95% CI)	*P*
WWI (continuous)	1.93 (1.67–2.14)	**< .001**	1.36 (1.19–1.56)	**< .001**	1.24 (1.09–1.41)	**.001**
WWI quartiles						
Q1	1.00 (Reference)		1.00 (Reference)		1.00 (Reference)	
Q2	2.33 (1.73–3.15)	**< .001**	1.30 (0.96–1.76)	.090	1.25 (0.91–1.71)	.163
Q3	3.21 (2.44–4.21)	**< .001**	1.40 (1.05–1.86)	**.022**	1.32 (0.99–1.76)	.057
Q4	4.34 (3.27–5.76)	**< .001**	1.62 (1.20–2.19)	**.002**	1.39 (1.03–1.89)	**.033**

Models were adjusted for the same covariates as in Table 2.

CI = confidence interval, CKD = chronic kidney disease, CVD = cardiovascular disease, HR = hazard ratio, WWI = weight-adjusted waist index.

### 3.3. Mediation analysis for NLR

Mediation models showed that WWI was positively associated with NLR (*β* = 0.10, 95% CI: 0.03–0.17, *P* = .004). After adjustment, WWI remained directly associated with all-cause (aHR = 1.17, 95% CI: 1.09–1.26, *P* < .001) and CVD mortality (aHR = 1.24, 95% CI: 1.09–1.41, *P* = .001). NLR independently predicted all-cause (aHR = 1.11, 95% CI: 1.08–1.15, *P* < .001) and CVD mortality (aHR = 1.09, 95% CI: 1.04–1.15, *P* < .001). The indirect effect of NLR accounted for 6.66% of the total effect on all-cause mortality (*β* = −9.94, 95% CI:−20.90–−2.85, *P* < .001) and 4.21% for CVD mortality (*β* = −30.37, 95% CI:−94.78–−4.41, *P* < .001) (Table 4 and 5). Thus, systemic inflammation partially mediated the link between central adiposity and adverse outcomes in CKD, though the direct pathway of WWI remained dominant.

**Table 4:** Mediation analysis of WWI, NLR, and all-cause mortality based on the fully adjusted Cox proportional hazards model.

Effect type	Estimate	95% CI (Lower–Upper)	*P* value	Mediation (%)
Indirect effect	−9.94	(−20.90–−2.85)	< .001	6.66
Direct effect	−145.40	(−305.74–−49.70)	< .001	93.34
Total effect	−155.35	(−320.57–−55.29)	< .001	100.00

Models were adjusted for the same covariates as in Table 2.

CI = confidence interval, NLR = neutrophil-to-lymphocyte ratio, WWI = weight-adjusted waist index.

**Table 5:** Mediation analysis of WWI, NLR, and CVD mortality based on the fully adjusted Cox proportional hazards model.

Effect type	Estimate	95% CI (Lower–Upper)	*P* value	Mediation (%)
Indirect effect	−30.37	(−94.78–−4.41)	< .001	4.21
Direct effect	−750.15	(−2335.59–−94.81)	< .001	95.79
Total effect	−780.52	(−2432.04–−100.43)	< .001	100.00

Models were adjusted for the same covariates as in Table 2.

CI = confidence interval, CVD = cardiovascular disease, NLR = neutrophil-to-lymphocyte ratio, WWI = weight-adjusted waist index.

## 4. Discussion

This large-scale cohort study using NHANES data aimed to investigate the association between the WWI (a novel obesity indicator) and all-cause and CVD mortality among 5582 patients with CKD, and to evaluate the mediating role of NLR. To our knowledge, this is the first study to explore the interrelationships among WWI, NLR and mortality. The principal findings were as follows. First, WWI showed an independent risk factor for all-cause and CVD mortality in CKD patients, and the relationship remained significant after adjusting for BMI, confirming the unique value of WWI beyond conventional obesity indicators. Second, NLR exerted a partial mediating effect on the WWI–mortality relationship, providing mechanistic insight into the “obesity–inflammation–mortality” pathway, despite its limited contribution. Third, subgroup analyses revealed important effect-modifying patterns, with the WWI-associated risk being more pronounced in men, younger participants, and those without hypertension, diabetes, or CVD, suggesting the heterogeneous risk of central obesity across clinical characteristics.

Obesity is a chronic, progressive, and relapsing condition characterized by excessive or abnormal fat accumulation.^[20]^ As a newer anthropometric index, WWI provides a more comprehensive reflection of overall fat distribution. Prior studies have shown that WC is associated with the incidence and prognosis of several diseases.^[14,21,22]^ Consistent with these findings, our study demonstrated that elevated WWI independently predicted both all-cause and CVD mortality among CKD patients. Individuals with higher WWI are more likely to experience adverse outcomes, which may be attributed to central adiposity–induced chronic low-grade inflammation, insulin resistance, and endothelial dysfunction that accelerate atherosclerosis and cardiovascular injury.^[23]^ Obesity is strongly associated with multiple comorbidities such as type 2 diabetes, hypertension, and dyslipidemia, all of which increase systemic oxidative stress and organ burden, thereby elevating mortality risk.^[24]^ In addition, obesity contributes to left ventricular hypertrophy, impaired cardiac reserve, and renal hemodynamic alterations, further predisposing individuals to CKD progression and fatal cardiovascular events.^[25]^ In addition to its biological plausibility, we further compared WWI with traditional anthropometric indices, including BMI and WC, to evaluate its incremental prognostic value. In Z-standardized Cox regression analyses, WWI demonstrated more stable and consistent associations with both all-cause and cardiovascular mortality compared with BMI and WC, which showed weaker or less consistent effects across models (Table S2, Supplemental Digital Content 3). Furthermore, model discrimination analysis using Harrell C-index suggested that WWI provided slightly improved predictive performance compared with traditional obesity indices. The improvement in C-index was modest. Notably, the combination of WWI and BMI yielded the highest C-index, indicating that WWI may provide complementary information beyond conventional anthropometric measures in risk stratification (Table S3, Supplemental Digital Content 4). NLR is a recognized marker of systemic inflammation.^[26]^ Inflammation plays a central role in the pathophysiological process of CKD.^[27]^ Obesity represents a state of chronic low-grade inflammation, often accompanied by elevated NLR, both serving as indicators of inflammatory activity.^[28]^ A multicenter study found that inflammatory mediators could bridge the association between obesity indices and poor outcomes in hypertensive patients.^[29]^ Chronic inflammation is considered the key link between obesity and multiple metabolic and cardiovascular disorders. During obesity, excessive expansion and dysfunction of adipose tissue lead to the release of proinflammatory cytokines such as interleukin‑6 and tumor necrosis factor-alpha, initiating persistent systemic inflammation.^[30,31]^ Numerous studies have documented that CKD patients exhibit immune dysregulation, hyperactivated inflammatory signaling, impaired anti-inflammatory responses, and sustained chronic inflammation.^[32–35]^ In a murine model of myocardial infarction, although N1 neutrophils predominated postinfarction, the proportion of N2 neutrophils increased from 2.4 ± 0.6% on day 1 to 18.1 ± 3.0% on day 7,^[36]^ indicating a transient surge in neutrophil counts during the first week after infarction. A cross-sectional study further revealed a significant reduction in total lymphocyte counts among end-stage renal disease patients compared with healthy controls.^[37]^ Recent evidence suggests that peripheral lymphocytes exert cytoprotective effects and contribute to improved cellular function.^[38,39]^ Conversely, circulating monocytes and neutrophils can upregulate matrix metalloproteinase-9 levels, thereby amplifying systemic inflammation.^[40,41]^ Moreover, neutrophils are reported to generate reactive oxygen species.^[40–42]^ potentially contributing to renal function decline and adverse outcomes. In summary, increased neutrophil counts combined with reduced lymphocytes may explain the observed elevation of NLR and its mediation of higher all-cause and CVD mortality risks in CKD patients. Based on these findings, we identified a significant interconnection among inflammation, obesity, and mortality in CKD. Our analysis revealed that NLR accounted for 6.66% and 4.21% of the mediating effects in all-cause and CVD mortality, respectively. Importantly, additional sensitivity analyses excluding participants with eGFR < 15 mL/minute/1.73 m^2^ and subgroup analyses across different baseline eGFR categories produced consistent findings, suggesting that the observed association between WWI and mortality was robust and not solely driven by patients with advanced CKD.

Although our study provides new insights into the association between WWI and mortality among CKD patients and reveals the potential role of inflammation, several limitations should be acknowledged. First, the cross-sectional nature of NHANES restricts causal inference, as it captures associations at a single time point. Second, due to data limitations, not all potential confounders could be adjusted for. Third, some clinical variables (such as medication use and self-reported disease history) were based on participants’ recollection rather than objective measures, introducing possible information bias. Finally, the relatively low proportion of CVD deaths among the 5582 participants might have introduced statistical uncertainty. Larger cohort studies with extended follow-up are warranted to validate our findings and strengthen the evidence base for the observed associations.

## 5. Conclusions

Elevated WWI was independently associated with higher risks of all-cause and CVD mortality among patients with CKD, and these associations were partially mediated by systemic inflammation reflected by NLR. Our findings highlight the importance of managing central obesity and inflammatory burden to improve prognosis in CKD populations.

## Author contributions

**Conceptualization:** Hao Zhou, Xue Zhang.

**Data curation:** Hao Zhou, Xue Zhang, Wanjun Zhang.

**Formal analysis:** Hao Zhou, Xue Zhang.

**Investigation:** Hao Zhou, Wanjun Zhang.

**Methodology:** Hao Zhou, Wanjun Zhang, Hairong Wang.

**Supervision:** Guanping Liu.

**Validation:** Xue Zhang, Guanping Liu.

**Writing – original draft:** Hao Zhou, Xue Zhang.

**Writing – review & editing:** Hao Zhou, Guanping Liu, Hairong Wang.








