# Supplementary material for: Weight-adjusted waist index and all-cause and cardiovascular mortality in chronic kidney disease: A cohort study with mediation analysis of systemic inflammation
Source: Medicine (Baltimore). 2026 Sep 4;105(36):e50613. doi: 10.1097/MD.0000000000050613 (PMC13549583; doi:10.1097/MD.0000000000050613)
Supplement: Supplementary file 1 [file medi-105-e50613-s002.docx]

**Table S1**. Sensitivity analyses of WWI and mortality in CKD

**A**. Excluding participants with eGFR <15 mL/min/1.73 m²

| Outcome | Model1 | |  | Model2 | |  | Model3 | |
| --- | --- | --- | --- | --- | --- | --- | --- | --- |
|  | HR (95%CI) | *P* |  | HR (95%CI) | *P* |  | HR (95%CI) | *P* |
| All-cause mortality | 1.79 (1.67 - 1.91) | **<.001** |  | 1.28 (1.19 - 1.38) | **<.001** |  | 1.17 (1.09 - 1.26) | **<.001** |
| CVD mortality | 1.95 (1.76 - 2.16) | **<.001** |  | 1.38 (1.20 - 1.59) | **<.001** |  | 1.25 (1.09 - 1.43) | **0.001** |
| Models were adjusted for the same covariates as in Table 2. | | | | | | | | |

Abbreviations: HR: Hazard Ratio; CI: Confidence Interval; WWI: Weight-Adjusted Waist Index; eGFR: Estimated Glomerular Filtration Rate; CVD: Cardiovascular Disease.

**B**. Full NHANES 1999–2018 cohort (extended follow-up)

| Outcome | Model1 | |  | Model2 | |  | Model3 | |
| --- | --- | --- | --- | --- | --- | --- | --- | --- |
|  | HR (95%CI) | *P* |  | HR (95%CI) | *P* |  | HR (95%CI) | *P* |
| All-cause mortality | 1.77 (1.66 - 1.89) | **<.001** |  | 1.28 (1.19 - 1.38) | **<.001** |  | 1.17 (1.09 - 1.26) | **<.001** |
| CVD mortality | 1.93 (1.74 - 2.14) | **<.001** |  | 1.37 (1.20 - 1.57) | **<.001** |  | 1.25 (1.09 - 1.43) | **0.001** |
| Models were adjusted for the same covariates as in Table 2. | | | | | | | | |

Abbreviations: HR: Hazard Ratio; CI: Confidence Interval; WWI: Weight-Adjusted Waist Index; eGFR: Estimated Glomerular Filtration Rate; CVD: Cardiovascular Disease.
