# Supplementary material for: Weight-adjusted waist index and all-cause and cardiovascular mortality in chronic kidney disease: A cohort study with mediation analysis of systemic inflammation
Source: Medicine (Baltimore). 2026 Sep 4;105(36):e50613. doi: 10.1097/MD.0000000000050613 (PMC13549583; doi:10.1097/MD.0000000000050613)
Supplement: Supplementary file 2 [file medi-105-e50613-s003.docx]

| Variables | Model1 | |  | Model2 | |  | Model3 | |
| --- | --- | --- | --- | --- | --- | --- | --- | --- |
|  | HR (95%CI) | *P* |  | HR (95%CI) | *P* |  | HR (95%CI) | *P* |
| BMI Z score | 0.84 (0.80 ~ 0.88) | **<.001** |  | 1.03 (0.98 ~ 1.08) | 0.251 |  | 0.93 (0.88 ~ 0.98) | **0.008** |
| WWI Z score | 1.44 (1.38 ~ 1.50) | **<.001** |  | 1.15 (1.10 ~ 1.21) | **<.001** |  | 1.07 (1.02 ~ 1.13) | **0.007** |
| WC Z score | 1.03 (0.99 ~ 1.07) | 0.188 |  | 1.08 (1.03 ~ 1.14) | **0.002** |  | 0.98 (0.93 ~ 1.03) | 0.454 |
| Model1: Crude | | | | | | | | |
| Model2:Adjust:Sex, Age,Race, Education,Marital status,PIR,Drinking,Smoking,Daily energy intake(2-day average). | | | | | | | | |
| Model3: Adjust: Sex, Age,Race, Education,Marital status,PIR,Drinking,Smoking,Daily energy intake(2-day average),HTN,DM,Hyperlipidemia,CVD,Serum albumin, Hb,uACR, eGFR. | | | | | | | | |

**Table S2**. Association of standardized anthropometric indices with mortality (Z-score Cox models)

**A**. All-cause mortality

**B**. Cardiovascular disease mortality

| Variables | Model1 | |  | Model2 | |  | Model3 | |
| --- | --- | --- | --- | --- | --- | --- | --- | --- |
|  | HR (95%CI) | *P* |  | HR (95%CI) | *P* |  | HR (95%CI) | *P* |
| BMI Z score | 0.87 (0.81 ~ 0.94) | **<.001** |  | 1.11 (1.02 ~ 1.21) | **0.012** |  | 1.00 (0.91 ~ 1.09) | 0.919 |
| WWI Z score | 1.49 (1.39 ~ 1.60) | **<.001** |  | 1.21 (1.12 ~ 1.31) | **<.001** |  | 1.11 (1.03 ~ 1.21) | **0.010** |
| WC Z score | 1.07 (1.01 ~ 1.15) | **0.045** |  | 1.16 (1.07 ~ 1.26) | **<.001** |  | 1.04 (0.95 ~ 1.14) | 0.380 |
| Models were adjusted for the same covariates as in Table S1A. | | | | | | | | |

Abbreviations: HR: Hazard Ratio; CI: Confidence Interval; BMI: Body Mass Index; WWI: Weight-Adjusted Waist Index; WC: Waist Circumference; HTN: Hypertension; DM: Diabetes Mellitus; CVD: Cardiovascular Disease; PIR: Poverty Income Ratio; eGFR: Estimated Glomerular Filtration Rate.
