# Supplementary material for: Weight-adjusted waist index and all-cause and cardiovascular mortality in chronic kidney disease: A cohort study with mediation analysis of systemic inflammation
Source: Medicine (Baltimore). 2026 Sep 4;105(36):e50613. doi: 10.1097/MD.0000000000050613 (PMC13549583; doi:10.1097/MD.0000000000050613)
Supplement: Supplementary file 3 [file medi-105-e50613-s004.docx]

**Table S3.** Model discrimination of anthropometric indices for mortality in CKD.

**A.** All-cause mortality

| **Model** | **C-index** | **SE** | **C-index (95% CI)** | **Description** |
| --- | --- | --- | --- | --- |
| Model 1 | 0.7786 | 0.00495 | 0.7689–0.7883 | Base model |
| Model 2 | 0.7792 | 0.00495 | 0.7695–0.7889 | WWI |
| Model 3 | 0.7792 | 0.00495 | 0.7695–0.7889 | BMI |
| Model 4 | 0.7787 | 0.00495 | 0.7689–0.7884 | WC |
| Model 5 | 0.7806 | 0.00494 | 0.7709–0.7903 | WWI + BMI |

Model: Sex, Age,Race, Education,Marital status,PIR,Drinking,Smoking,Daily energy intake(2-day average),HTN,DM,Hyperlipidemia,CVD,Serum albumin, Hb,uACR, eGFR.

Abbreviations: HR: Hazard Ratio; CI: Confidence Interval; WWI: Weight-Adjusted Waist Index; BMI: Body Mass Index; WC: Waist Circumference; HTN: Hypertension; DM: Diabetes Mellitus; CVD: Cardiovascular Disease; PIR: Poverty-Income Ratio; Hb: Hemoglobin; uACR: Urinary Albumin-to-Creatinine Ratio; eGFR: Estimated Glomerular Filtration Rate; SE: Standard Error.

**B.** Cardiovascular disease mortality

| **Model** | **C-index** | **SE** | **C-index (95% CI)** | **Description** |
| --- | --- | --- | --- | --- |
| Model 1 | 0.8015 | 0.00727 | 0.7872–0.8157 | Base model |
| Model 2 | 0.8027 | 0.00725 | 0.7885–0.8169 | WWI |
| Model 3 | 0.8015 | 0.00727 | 0.7872–0.8158 | BMI |
| Model 4 | 0.8015 | 0.00727 | 0.7873–0.8158 | WC |
| Model 5 | 0.8030 | 0.00725 | 0.7888–0.8172 | WWI + BMI |

Model: Sex, Age,Race, Education,Marital status,PIR,Drinking,Smoking,Daily energy intake(2-day average),HTN,DM,Hyperlipidemia,CVD,Serum albumin, Hb,uACR, eGFR.

Abbreviations: HR: Hazard Ratio; CI: Confidence Interval; WWI: Weight-Adjusted Waist Index; BMI: Body Mass Index; WC: Waist Circumference; HTN: Hypertension; DM: Diabetes Mellitus; CVD: Cardiovascular Disease; PIR: Poverty-Income Ratio; Hb: Hemoglobin; uACR: Urinary Albumin-to-Creatinine Ratio; eGFR: Estimated Glomerular Filtration Rate; SE: Standard Error.
